# Bioactive Compounds and Their Impact on Protein Modification in Human Cells

**DOI:** 10.3390/ijms23137424

**Published:** 2022-07-04

**Authors:** Ankush Prasad, Claudio Rossi, Renuka Ramalingam Manoharan, Michaela Sedlářová, Lorenzo Cangeloni, Deepak Rathi, Gabriella Tamasi, Pavel Pospíšil, Marco Consumi

**Affiliations:** 1 Department of Biophysics, Faculty of Science, Palacký University, Šlechtitelů 27, 783 71 Olomouc, Czech Republic; renuka.rmanoharan@gmail.com (R.R.M.); deepak1234rathi@gmail.com (D.R.); pavel.pospisil@upol.cz (P.P.); 2 Department of Biotechnology, Chemistry and Pharmacy, University of Siena, Via Aldo Moro 2, 53100 Siena, Italy; cangeloni@student.unisi.it (L.C.); gabriella.tamasi@unisi.it (G.T.); marco.consumi@unisi.it (M.C.); 3 Department of Botany, Faculty of Science, Palacký University, Šlechtitelů 27, 783 71 Olomouc, Czech Republic; michaela.sedlarova@upol.cz

**Keywords:** bioactive compounds, antioxidants, nutraceuticals, monocyte, macrophage, reactive oxygen species, phorbol 12-myristate 13-acetate, protein modification, malondialdehyde, redox reactions

## Abstract

Reactive oxygen species (ROS) represent a group of molecules with a signaling role that are involved in regulating human cell proliferation and differentiation. Increased ROS concentrations are often associated with the local nonspecific oxidation of biological macromolecules, especially proteins and lipids. Free radicals, in general, may randomly damage protein molecules through the formation of protein-centered radicals as intermediates that, in turn, decay into several end oxidation products. Malondialdehyde (MDA), a marker of free-radical-mediated lipid oxidation and cell membrane damage, forms adducts with proteins in a nonspecific manner, leading to the loss of their function. In our study, we utilized U-937 cells as a model system to unveil the effect of four selected bioactive compounds (chlorogenic acid, oleuropein, tomatine, and tyrosol) to reduce oxidative stress associated with adduct formation in differentiating cells. The purity of the compounds under study was confirmed by an HPLC analysis. The cellular integrity and changes in the morphology of differentiated U-937 cells were confirmed with confocal microscopy, and no significant toxicity was found in the presence of bioactive compounds. From the Western blot analysis, a reduction in the MDA adduct formation was observed in cells treated with compounds that underlaid the beneficial effects of the compounds tested.

## 1. Introduction

Nutrients that are not synthesized inside the human body but supplied with the diet have potential health benefits by affecting our microbiome; they are important for the metabolism, immunity, and protection of cells/organs from metabolic or systematic byproducts, for instance, free radicals and inflammatory molecules that can otherwise be harmful if exceeded beyond the scavenging limit of the non-enzymatic or enzymatic defense system [1,2,3]. The wide range of such molecules includes both plant and animal origins, e.g., polyphenols, carotenoids, vitamins, omega-3 fatty acids, organic acids, amino acid derivatives, etc. [4,5,6]. These natural compounds are being studied for their role in the alteration in biological reactions and their activity as antioxidant, anti-inflammatory, stress-protective, anticancer, cardioprotective, and neuroprotective agents. In the present study, we selected four bioactive compounds, i.e., chlorogenic acid, oleuropein, tomatine, and tyrosol, of which oleuropein and tomatine were extracted from their natural sources (olive and tomato leaves, respectively). Chlorogenic acid and tyrosol were commercial products. Their role as anti-inflammatory and antioxidant agents in vitro systems was evaluated.

Among antioxidants, chlorogenic acid is one of the polyphenols present in plants, abundantly consumed with fruits, vegetables, coffee, and tea. It is formed from the esterification of quinic acid and certain trans-cinnamic acids, most commonly caffeic, *p*-coumaric, and ferulic acids [7]. Chlorogenic acids are characterized by different subgroups consisting of caffeoylquinic, *p*-coumaroylquinic, and feruloyquinic acids. These acids are abundant at different concentrations and in isomeric mixtures in drinking beverages such as coffee, tea, and wine [7]. In published studies, beverages such as coffee and various fruits that contained these acids lowered the risk of some chronic diseases with their antioxidant, anti-inflammatory, and antibacterial properties [8,9]. A study on a rat model in a gastric epithelial model showed that chlorogenic acids are not hydrolyzed in the gastrointestinal tract and are actively absorbed [10]. Chlorogenic acid and its microbial metabolites were proposed to decrease the proliferation of colon cancer cells [11].

Tomatine, a glycoalkaloid with antibiotic and insecticide effects, can be extracted from the stems, leaves, and green unripe tomatoes (*Solanum lycopersicum* L.); it represents one of the main parts of the Mediterranean diet, which is known to contain other antioxidants, phenolic compounds, etc. [12]. In recent studies, the extracts obtained from green unripe tomatoes have been shown to have antihypertensive [13], anticancer, and antimicrobial properties that induce the cell death rate of cancer cells, which have been studied in different cancer cell lines [14,15]. On the contrary, the direct effects of tomatine or tomato extract on protein modification in any in vivo experiments have not been studied.

α-tomatine was found to be the most effective agent against cancer cell growth. In tomato extract upon in vitro digestion (condition as in vivo gastrointestinal environment), around 45–50% antioxidant activity was still retained [16]. The compound is also known to balance the level of free radicals in cells and control oxidative damage [17,18,19]. The cell toxicity, antioxidants, and other physiological effects of tomatine depend on the ripening stage [20,21].

Oleuropein can be extracted from olive leaves (*Olea europaea* L.). It consists of a molecule of elenolic acid linked to orthodiphenol hydroxytyrosol by an ester bond, and to a molecule of glucose by a glycosidic bond extracted from olive leaves, and is one of the main phenolic secoiridoids [22]. This compound has well-known anti-inflammatory and antioxidant effects, as evidenced in different studies [23,24,25,26]. The effect of oleuropein was studied in rats, and the oxidative stress and antioxidant activity were observed by the evaluation of the levels of malondialdehyde (MDA), superoxide dismutase (SOD), catalase (CAT), glutathione peroxidase (GPX), myeloperoxidase (MPO), and nitric oxide (NO). Different anti-inflammatory genes and cytokines such as IL-1β, TNF-α, IL-10, COX-2, iNOS, TGF-β1, MCP-1, and NF-κB, the pro-apoptotic gene Bax, and the antiapoptotic gene Bcl2 were also evaluated in rat tissue and found altered [22]. After oleuropein treatment, the MDA, MPO, and NO levels were significantly lower, and the levels of SOD, CAT, and GPX were elevated [22].

Tyrosol, a derivative of phenethyl alcohol, is a phenylethanoid. The main sources of tyrosol are olive leaves, and olive oil can form esters with fatty acids [27]. In clinical studies, tyrosol exerted protective effects against oxidative damage caused by oxidized LDL in the adenocarcinoma cell line [28]. It was also suggested that tyrosol may neutralize cellular damage induced by reactive oxygen metabolites. Studies on the effect of tyrosol on levels of superoxide anion radicals (O_2_^●−^), hydrogen peroxide (H_2_O_2_), and nitric oxide (^●^NO) were studied in stimulated macrophages with ascorbic acid and phorbol esters, where tyrosol showed a negative effect on O_2_^●−^ and H_2_O_2_ production [29]. Tyrosol is also known to modulate NF-κB activation, which plays a very important role in inflammatory reactions. Chemically synthesized tyrosol sulfate (Tyr-SUL) and tyrosol glucuronate (Tyr-GLU) and their metabolites were also studied for their anti-inflammatory and antioxidant activities; tyrosol inhibits ROS level rise and downregulates the expressions of glutathione peroxidase 1, glutamate-cysteine ligase catalytic subunit, and heme oxygenase-1 genes [30]. This investigation was carried out to evaluate the potential of these compounds for the prevention of protein modification, and tests were performed on a cell line.

The U-937 cell line we used in our study is a human pro-monocytic myeloid leukemia cell line [31]. A recent study revealed their differentiation behavior and their relationship to ROS production under the effect of inducers [32]. The advantage of using U-937 cells is that they do not succumb to the Hayflick limit [33,34]. In the current study, phorbol 12 myristate 13-acetate (PMA) was used as a differentiation inducer. The differentiation depends on the inducer, its concentration, and the length of incubation [35]. Induction of the differentiation by PMA occurs through the activation of protein kinase C (PKC) isozymes, and the subsequent generations of ROS are known to be closely interlinked. More specifically, O_2_^●−^ is generated by nicotinamide adenine dinucleotide phosphate hydrogen (NADPH) oxidase, which can later be converted to H_2_O_2_ by SOD or subsequently to hydroxyl radical (HO^●^) in the presence of transition metal ions. In human cells, NADPH oxidase is primarily responsible for producing the ROS needed for redox signaling; various growth factors and cytokines stimulate ROS production by activating this enzyme [36]. Although it is unclear whether the ROS produced by the mitochondria contributes to redox signaling, it is becoming increasingly clear that the H_2_O_2_ released into the cytosol plays a role in various signaling networks, including cell cycle transition and redox balance [37]. Highly reactive species generated under normal physiological and oxidative stress conditions are able to directly oxidize proteins or through lipid derivatives that react with protein functional groups. These protein carbonyl derivatives serve as markers in elucidating ROS-mediated protein oxidation events. Malondialdehyde, an aldehyde derivative, forms adducts with proteins, thereby leading to a loss in protein function. In the present study, we focused on evaluating bioactive compounds in their ability to reduce the impact of ROS-mediated protein modification using U-937 as a cell line model. The differentiation of U-937 cells under the PMA induction was visualized by confocal laser scanning microscopy (CLSM), with a focus on the plasma membrane and nuclei integrity. Subsequently, the effect of four bioactive compounds (chlorogenic acid, oleuropein, tomatine, and tyrosol) on U-937 cells was evaluated using the immunoblotting technique to understand protein modifications.

## 2. Results and Discussion

### 2.1. Characterization of Bioactive Compounds

**Figure 1:**
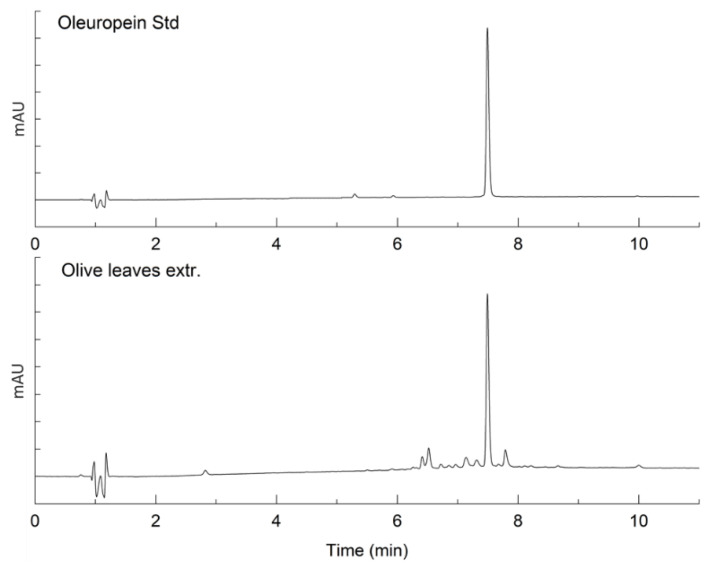
Stacked UV chromatogram of the oleuropein standard and the olive leaf extract.

**Figure 2:**
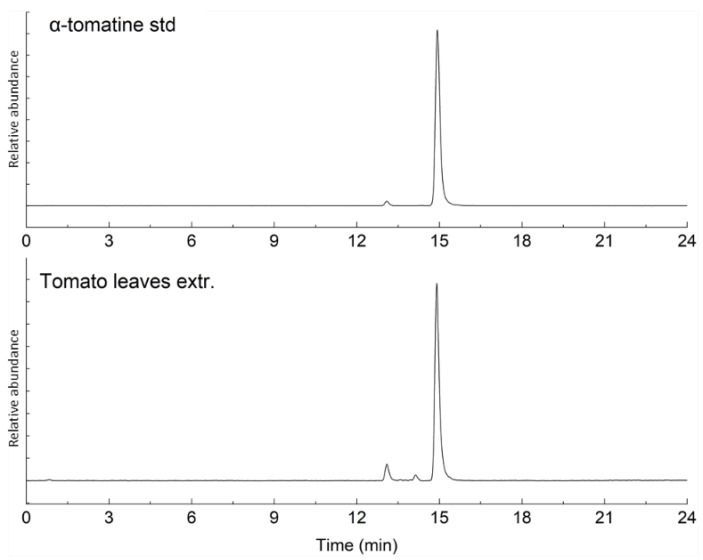
Stacked mass chromatograms of α-tomatine standard and the tomato leaf extract.

To characterize the oleuropein and α-tomatine originating in the extracts from olive and tomato leaves, HPLC-UV and HPLC-MS analyses were performed, respectively. In Figure 1, the UV chromatograms of the oleuropein standard and the olive leaves extract are reported. Figure 2 shows the stacked mass chromatograms of the α-tomatine standard and tomato leaf extract. The percent purity of the extracted compounds was evaluated on a calibration curve and is presented in Table 1. Other peaks observed in the chromatograms of the tomato leaves extracts were related to minor glycoalkaloids, dehydrotomatine, and β-tomatine. In the olive leaves extracts, the other compounds were hydroxytyrosol, some flavonoids (luteolin and apigenin glycosides), and the phenylpropanoid verbascose.

### 2.2. Cell Viability Using MTT Assay

**Figure 3:**
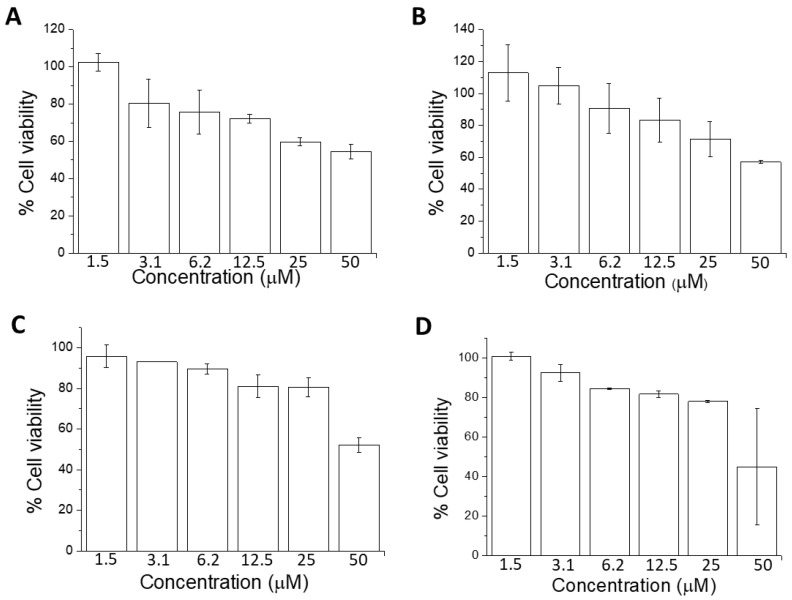
Cell viability of U-937 cells. PMA-treated U-937 cells with chlorogenic acid (**A**), oleuropein (**B**), α-tomatine (**C**), and tyrosol (**D**) at different concentrations. Data are presented as mean ± SE.

Quantitative estimation of viable cells exposed to bioactive compounds in U-937 cells was carried out using an MTT assay. U-937 cells were treated with PMA along with bioactive compounds for 72 h. From Figure 3, the cell viability under different concentrations of bioactive compounds used in the experiments exceeded 70%. Prior to the assay, differentiation of cells was induced with 250 nM PMA, a concentration optimized in our previous study [38]. The effect of bioactive compounds applied at several concentrations was dose-dependent; in Figure 3, it is expressed using PMA as 100% control. With increasing concentrations (1.5, 3.1, 6.2, 12.5, 25, and 50 μM), the percentage of viable cells decreased linearly, but was found to be always higher than 70% in the concentration used in our study. Chlorogenic acid (Figure 3A) had the strongest effect on cell viability compared with oleuropein (Figure 3B), tomatine (Figure 3C), and tyrosol (Figure 3D). The percentage viability in the case of chlorogenic acid was ~70%, while in the case of the others, it was well above 80%. As mentioned above, cell viability corresponds to metabolically active cells in response to treatments. The decrease in cell viability was evident for concentrations above 12.5 μM; therefore, we utilized 10 μM or lower as the standard concentration for treatment. The results were further validated using the trypan blue exclusion test (Supplementary Data S1).

### 2.3. U-937 Cell Morphology

**Figure 4:**
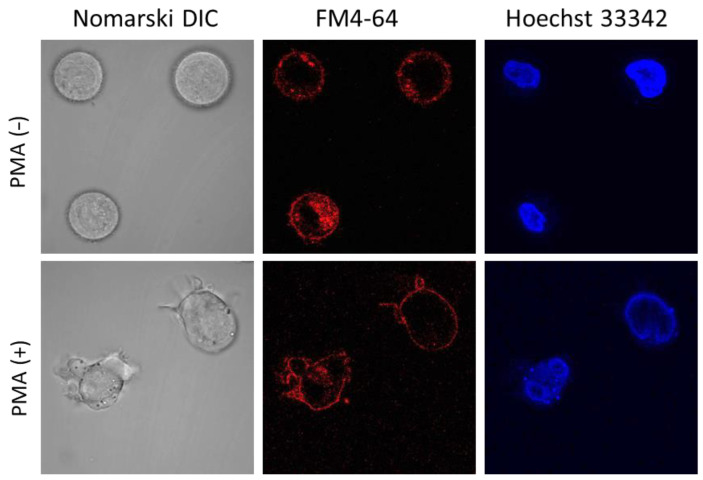
Double staining using FM4-64 and Hoechst 33342 in nondifferentiated (upper panel) and 72 h differentiated (lower panel) U-937 cells incubated for 5 min. Images (magnification 1000×) were taken in different channels (from left to right are Nomarski DIC, FM4-64, and Hoechst 33342).

**Figure 5:**
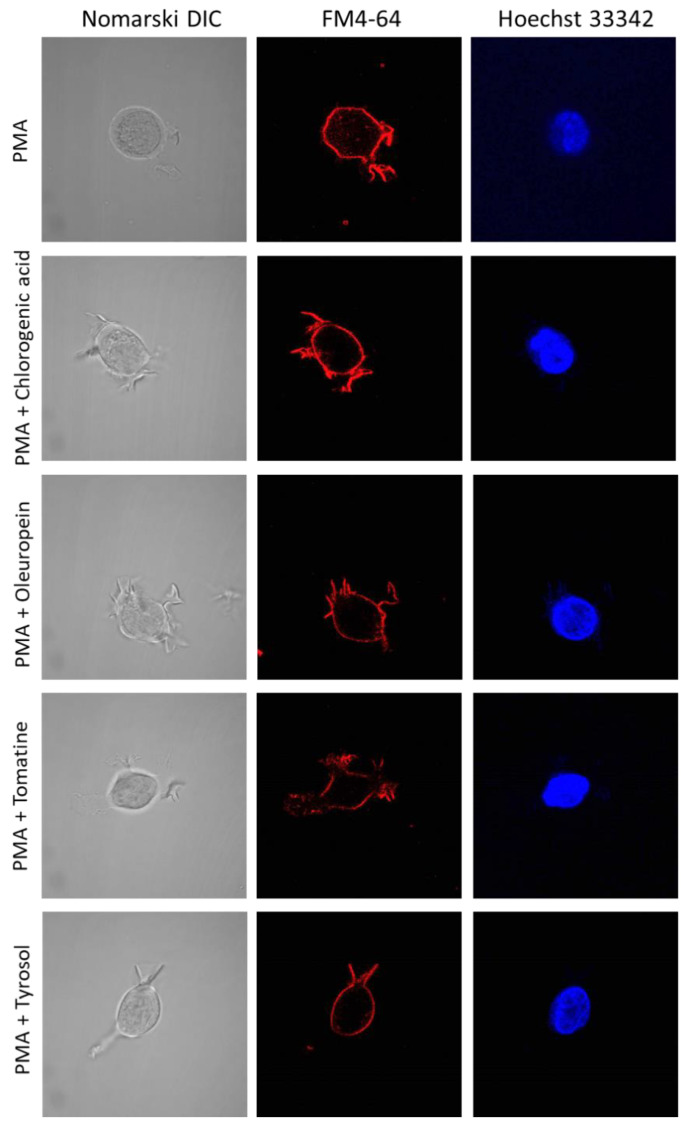
Double staining using Hoechst 33342 and FM4-64 in 72 h differentiated U-937 cells and in the presence of either of the bioactive compounds (chlorogenic acid, oleuropein, α-tomatine, and tyrosol, 10 μM). The staining was performed for 5 min, and images (magnification 1000×) were taken in different channels (from left to right are Nomarski DIC, FM4-64 and Hoechst 33342).

Cell morphology was monitored using CLSM following incubation with 250 nm PMA (Figure 4) for 72 h, and a high percentage of induction was observed. Differentiated U-937 cells have a distinct extension bearing amoeboid morphology, which confirms the maturation of promonocytic cells into monocytes or macrophages. Control cells (−PMA, upper panel) showed a mainly spherical, translucent structure, while most treated cells (+PMA, lower panel) formed pseudopodia. In U-937 cells treated with PMA in the absence or presence of bioactive compounds (Figure 5 and Supplementary Data S2), cellular integrity was maintained both for the plasma (middle panel) and nuclear (right panel) membranes. Figure 4 and Figure 5 and Supplementary Data S2 present multiple observation and scans performed on biological and technical replicates.

When cells such as U-937 cells are exposed to inducers such as PMA, their proliferation slows while the differentiation process is triggered. The monocytes under the PMA treatment are known as “macrophage-like” because of their structure [39,40,41]. However, the properties of the transformed cell line are not yet well-known. Phorbol 12-myristate 13-acetate works as a tumor promoter, and it is known to be involved in gene transcription, cell growth, differentiation, immune pathway, programmed cell death, and receptor desensitization through PKC signaling pathways [42]. It also starts to induce adherence accompanied by cell cycle arrest [43]. PMA is also known to activate the calcium- and phospholipid-dependent isoforms of PKC, thus inducing AMP metabolism, leading to its maturation into a macrophage. It is now well-accepted that adding PMA exogenously activates the NADPH oxidase complex, which can lead to the formation of O_2_^●−^. In the presence of SOD, it can create H_2_O_2_ and subsequently HO^●^ [3,43,44,45,46].

### 2.4. Protein Modification in Differentiating Cells and Impact of Bioactive Compounds

**Figure 6:**
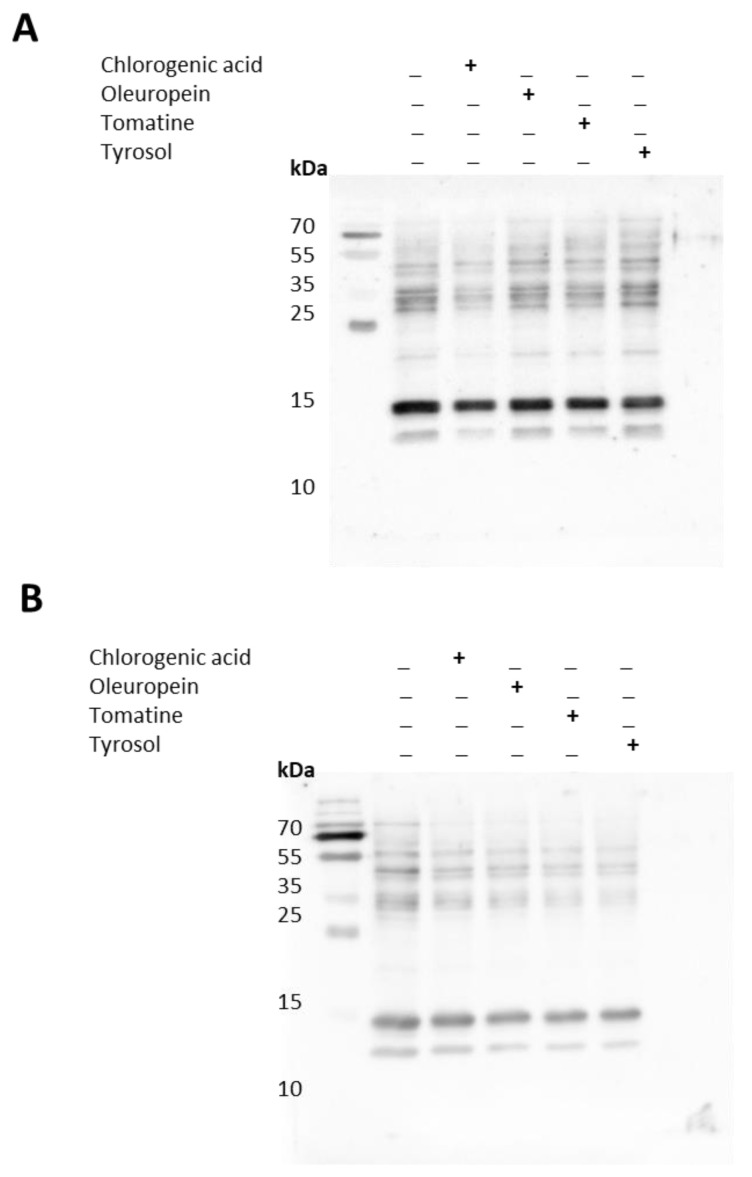
Identification of protein MDA adducts in whole-cell homogenates of U-937 cells treated with bioactive compounds PMA or PMA + bioactive compounds. Anti-MDA blot represents protein modification in U-937 cells differentiated for 48 h (**A**) and 72 h (**B**).

**Figure 7:**
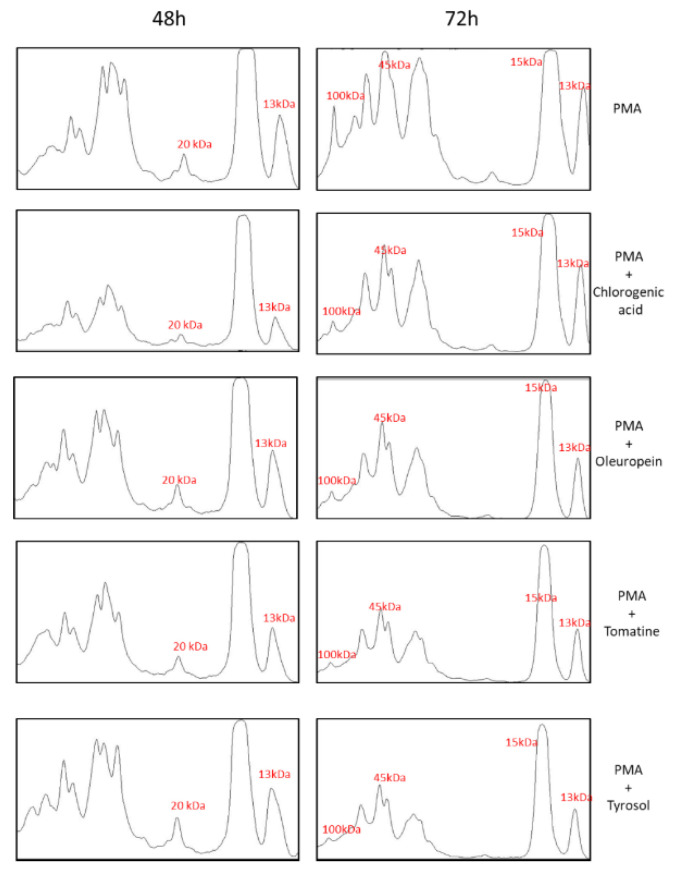
Quantification of protein bands from an anti-MDA blot (Figure 6) by densitogram analysis is shown and selected proteins are indicated with respective molecular weight (red).

The whole-cell homogenate of U-937 cells treated with PMA alone (48 and 72 h) or together with bioactive compounds (24 h) was separated using SDS-PAGE. Immunoblotting using anti-MDA antibodies showed a modification of more than one protein. In 48 h differentiated cells (with last 24 h in the presence of bioactive compounds), the MDA was most evident in the protein band, corresponding approximately to 13 and 20 kDa (Figure 6A and Figure 7). On the densitogram presented as a part of Figure 7, it can be clearly seen that there was a significant suppression in the bands mentioned above. In the 72 h differentiated cells (with last 24 h in the presence of bioactive compounds), significant suppression in the bands at 13, 15, 45, and 100 kDa was seen. α-tomatine showed the maximum antioxidant capacity compared with chlorogenic acid in the 72 h differentiated cells (Figure 6B and Figure 7).

**Figure 8:**
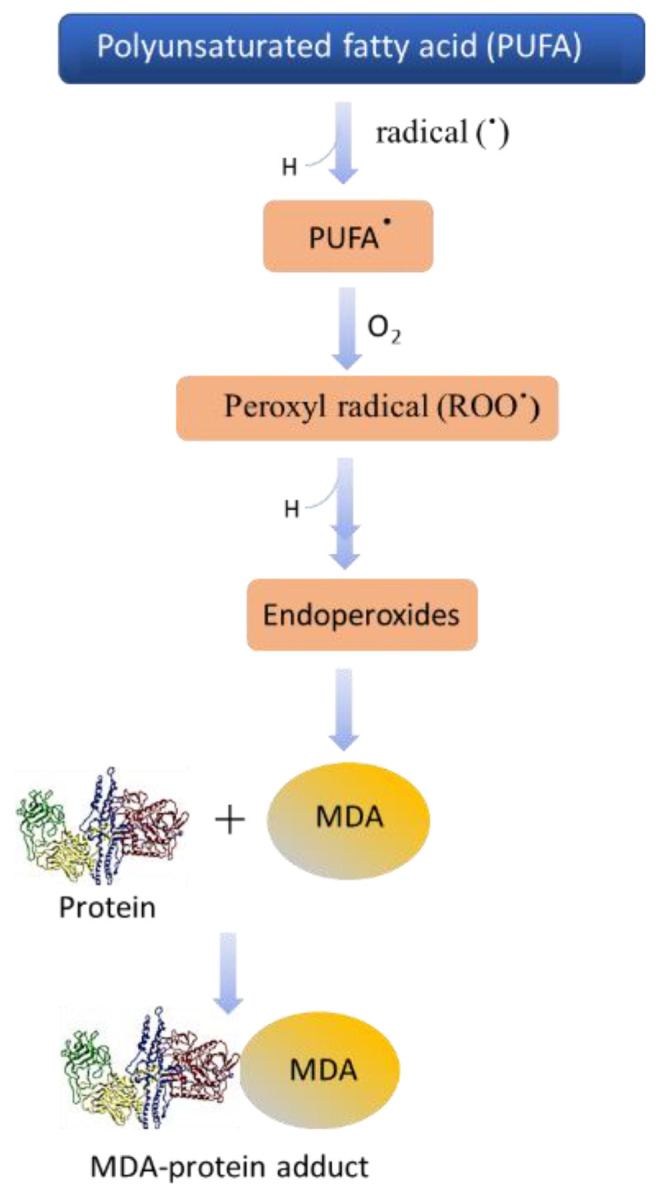
Schematic representation showing the steps involved in protein modification as the results of ROS generation and successive oxidative radical reactions.

Under oxidative stress, the plasma membrane lipids of cells can undergo oxidation that indirectly results in HO^●^-mediated protein carboxylation [47]. Polyunsaturated acyl chains of phospholipids, arachidonic acid and linoleic acid (polyunsaturated fatty acids), are extremely prone to peroxidation and breakdown through nonenzymatic hock cleavage, which forms a different kind of lipid-derived aldehydes and ketones [48]. These lipid-derived aldehydes and ketones can diffuse into the cell and modify proteins in the cells. In previous studies, it was suggested that lipid-derived aldehydes are more reactive than direct oxidation of the amino acid side chain formed from protein carbonylation [49]. The scheme presented in Figure 8 shows the different steps involved in the protein modification as a result of ROS generation and successive oxidative radical reactions. Reactive oxygen species are important metabolic products of cells, and play a beneficial role in cell defense, but their reaction with other proteins can be harmful [37]. The main protein modification is sulfoxide, which is the result of oxidation of methionine, cysteine to sulfenic, sulfinic, and sulfonic acids due to different redox reactions. Hydrogen peroxide, for instance, can cause the oxidation of the side chain of protein amino acids, which results in the formation of semialdehyde amino acids; most reactions occur with lysine, arginine, and proline [50].

## 3. Methods and Materials

### 3.1. Extracts

Oleuropein extract was prepared from the leaves of olives (*Olea europaea* L.) by following the protocol of Tamasi et al., and the tomatine was obtained from the leaves of tomatoes (*Solanum lycopersicum* L.). The extraction protocols were previously reported for tomatine [21,51,52]. In brief, lyophilized leaves were treated with a hydroalcoholic mixture (EtOH/H_2_O; 80:20 *v*/*v*). For tomatine extraction, the mixture was acidified with acetic acid (1%, *v*/*v*). The suspension was extracted through an ultrasonic-assisted procedure to enhance the extraction process, then it was centrifuged, and the liquid fraction was collected. The extraction procedure was repeated twice on the remaining solid residue. Finally, the extracts were dried under a nitrogen stream, freeze-dried, and stored at −20 ± 1 °C, in dim-light conditions, before further analysis. All samples were extracted in triplicate. Chlorogenic acid (purity > 97%) and tyrosol (purity > 98%) were commercial products and were used without extra purification.

### 3.2. Reagents and Antibodies

All the solvents were purchased from Sigma-Aldrich (Milan, Italy): ethanol (EtOH, gradient grade for HPLC, ≥99.9%), methanol (MeOH, gradient grade for HPLC, ≥99.9%), acetonitrile (ACN, LC-MS grade, ≥99.9%), water (H_2_O, LC-MS grade, ≥99.9%), acetic acid (CH_3_COOH, ≥98%), and formic acid (HCOOH, LC-MS grade, ≥98.5%). The oleuropein standard was purchased from Sigma-Aldrich (Milan, Italy) (98%), and the tomatine standard was purchased from Extrasynthese (Lyon, France) (≥98.5%). Cell culture media and antibiotics were purchased from Biosera (Nuaille, France). Phorbol 12-myristate 13-acetate (PMA) was obtained from Sigma-Aldrich (St. Louis, MO, USA). The rabbit polyclonal anti-malondialdehyde (MDA) antibody and MTT cell proliferation assay kit were purchased from Abcam [anti-malondialdehyde (MDA) antibody (ab27642)] (Cambridge, UK). Polyclonal goat anti-rabbit IgG conjugated with horseradish peroxidase (HRP) was obtained from Bio-Rad (Hercules, CA, USA). Protease and phosphatase inhibitors were obtained from Roche (Mannheim, Germany).

### 3.3. HPLC-MS Analysis for α-Tomatine Determination in Tomato Leaf Extracts

The extracts of tomato leaves were analyzed to determine the content of α-tomatine by HPLC-MS analysis. The extracts were resuspended in methanol (MeOH), and the analysis was performed on an UltiMate 3000 HPLC coupled with an LTQ XL mass spectrometer equipped with a HESI II electrospray ion source. Xcalibur software was used to acquire and process the data. The determination of the α-tomatine content was performed following a method previously optimized and published, with some slight modifications [21,51]. A Phenomenex Kinetex Polar C18 (150 × 2.1 mm, 2.6 µm, 100 Å) with a SecurityGuard C18 guard column (2 × 2.1 mm), thermostated at 35 ± 1 °C, was used for the chromatographic separation. The eluents were H_2_O containing 0.1% formic acid (A) and acetonitrile containing 0.1% formic acid (B). The gradients were: isocratic 20% B (0–1 min), curve gradient 20–50% B curve parameter: 8 (1–23 min), and linear gradient 50–95% B (23–25 min). The injection volume was 3 μL, and the flow rate was kept at 0.4 mL/min. The electrospray parameters were optimized by direct injection of a tomatine standard diluted in the elution solvent composition in positive mode: spray voltage, 5 kV; sheath and auxiliary gas, 35 and 10 arbitrary units, respectively; the capillary temperature was maintained at 200 °C. The quantitative determination of α-tomatine was carried out in SIM mode via external calibration, using peimine as the internal standard. The same amount of internal standard (IS, 1.0 mg/L in MeOH) was added to all standards and on the samples (analyzed on the same day). The calibration curve was acquired by injecting standard solutions in the linearity range of concentrations, 0.025–10 mg/L, obtaining the equation y = 0.36693x, R^2^ = 0.9965. The limit of detection (LOD) and limit of quantification (LOQ) values were also determined as 0.007 and 0.020 mg/L, respectively. All samples were extracted and analyzed in triplicate (n = 9), and results are expressed in milligrams per gram as mean concentration ± standard deviation.

### 3.4. HPLC-UV Analysis for Oleuropein Determination in Olive Leaf Extracts

Olive leaf extracts were analyzed to determine the content of oleuropein by HPLC-UV analysis using a previous method with slight modifications [52]. The extracts were reconstituted in MeOH, and HPLC-UV analyses were performed on an UltiMate 3000 instrument equipped with an RS-3000 Diode Array Detector operating at 280 nm. Xcalibur software was used to acquire and process the data. A Kinetex Polar C18 column (150 × 2.1 mm, 2.6 µm, 100 Å; Phenomenex, Bologna, Italy) with a SecurityGuard C18 guard column (2 × 2.1 mm), thermostated at 35 ± 1 °C, was used for the chromatographic separation. The solvents used for the elution gradient were A (H_2_O containing 0.1% formic acid) and B (acetonitrile containing 0.1% formic acid); the linear gradient was eluted from 5% to 50% of B (0–10 min), and from 50% to 95% of B (10–15 min). The injection volume was 3 μL, and the flow rate of 0.4 mL/min. The quantitative determination of oleuropein was carried out via external calibration. The calibration curve was acquired by injecting standard solutions in the linearity range of concentrations, 5–100 mg/L, obtaining the equation y = 1930.8x, R^2^ = 0.9998. The LOD and LOQ were also determined as 1.00 and 3.00 mg/L, respectively. All samples were extracted and analyzed in triplicate (n = 9), and the results are expressed in milligrams per gram as mean concentration ± standard deviation.

### 3.5. Cell Line Origin and Cultivation

The U-937 cell line was obtained from the American Type Culture Collection (ATCC; Rockville, MD, USA). Cells were grown in RPMI-1640 supplemented with 0.05 mM L-glutamine, 10% fetal bovine serum (FBS), and 1% antibiotics (penicillin and streptomycin) in the *v*/*v* ratio. Experiments were carried out when viability was close to or above 70%. An automated cell counter (Bio-Rad Laboratories, Hercules, CA, USA) was used to determine cell density, and viability was monitored using 0.05% trypan blue dye.

### 3.6. Cell Line and Differentiation Condition

For experiments, cells were differentiated with 250 nM PMA at two different time points of 48 and 72 h. Following differentiation, cells were incubated with the bioactive compounds mentioned above for 24 h. All experiments were carried out in complete medium. A cell density of 1 × 10^6^ CFU/mL was used for treatment.

### 3.7. MTT Cell Proliferation Assay

Cell viability was determined using a Cell Proliferation Assay Kit (ab211091) (Sigma Aldrich GmbH, Mannheim, Germany). For the test, U-937 cells were seeded in replicates (*n* = 2) on a 96-well microplate and treated with PMA in the presence or absence of bioactive compounds (10 μM) at 37 °C. Followed by incubation, the media were discarded, and serum-free medium was added along with MTT reagent and incubated for 3 h at 37 °C. To avoid interference by the MTT reagent, MTT solvent was added, and the 96-well microplate was kept on the shaker for 15 min. The absorbance was recorded at 590 nm, and the results are presented as percentage of the control in Section 2.2. Data are expressed as ±SEM of at least two measurements.

### 3.8. Confocal Laser Scanning Microscopy

A Fluorview 1000 confocal unit attached to the IX80 microscope was utilized to visualize U-937 cells on slides (Olympus Czech Group, Prague, Czech Republic). Staining with FM4-64 (15 μM) and Hoechst 33342 (2 μM) for 5 min at RT was used to monitor the integrity of cell membranes and nuclei under experimental conditions. A lipophilic dye FM4-64 (Sigma Aldrich GmbH, Mannheim, Germany) bound to the plasma membrane was excited using a 543 nm He-Ne laser, and its emission was recorded within the range of 655–755 nm. Hoechst 33342 is a membrane-permeant stain specific for AT-rich regions of double-stranded DNA for which excitation was achieved by a 405 nm diode laser, and the signal was recorded with 430–470 nm bandpass filter.

### 3.9. Protein Immunoblotting

After differentiation and incubation with the bioactive compounds, U-937 cells were collected by centrifugation and washed with PBS (pH 7.4) to remove residual media. Cell pellets thus obtained were resuspended in lysis buffer(150 mM NaCl, 50 mM Tris (pH 8.0), 0.5% sodium deoxycholate, 0.1% SDS, and 1% NP-40) containing 1% (*v*/*v*) protease and phosphatase inhibitor (*v*/*v*) and subjected to sonication. The processed homogenate was centrifuged at 14,000× *g*, and the collected supernatant fraction was quantified using a Pierce BCA protein estimation kit (Thermo Fisher Scientific, Paisley, UK). Protein samples were prepared with 5× Laemmli sample buffer along with 100 mM 2-Mercaptoethanol, and a concentration of 10 μg/lane was used for electrophoresis.

Whole-cell homogenates separated on 10% SDS gel were then transferred to nitrocellulose membranes using a Trans-Blot Turbo transfer system (Bio-Rad, Hercules, CA, USA). Nitrocellulose membranes were then blocked for 2 h at RT with 5% BSA in phosphate-buffered saline (PBS) (pH 7.4) containing 0.1% Tween 20. The blocked membranes were probed overnight with an anti-MDA antibody at 4 °C and incubated for 1 h at room temperature with HRP-conjugated anti-rabbit secondary antibody (dilution 1:10,000). Immunocomplexes thus formed were visualized using Immobilon Western Chemiluminescent HRP Substrate (Sigma Aldrich, GmbH, Mannheim, Germany) and captured with an Amersham 600 imager (GE Healthcare, Amersham, UK).

## 4. Conclusions

Activation or differentiation of monocytes into macrophages is mediated by the expression of various proinflammatory mediators and the generation of O_2_^●−^. Lipids, more specifically polyunsaturated fatty acids, are highly vulnerable to oxidative stress. The compounds evaluated in the present study exhibited less cytotoxicity. Additionally, they do not interfere with the process of U-937 differentiation toward monocytic lineages, as confirmed by confocal analysis. Malondialdehyde, a byproduct of lipid peroxidation, is toxic, and its enhanced expression is known to be involved in various pathogenesis, including carcinogenesis, diabetics, and neurological disorders. It is a highly reactive molecule, with three carbon dialdehyde molecules that interact with functional groups on proteins, forming adducts. Immunoblotting analysis with anti-MDA antibody revealed that among the tested compounds, tomatine and tyrosol exhibited enhanced antioxidant activity compared with chlorogenic acid and oleuropein. A further transcriptomic analysis of U-937 after differentiation and incubation with antioxidants is required, which will help in the identification of the genes involved and their upregulation before and after antioxidant treatment.

## Figures and Tables

**Table 1:** The concentration of main components in the tomato and olive leaf extracts (*n* = 9).

Compound	Average Concentration (mg/g)	Standard Deviation	% RSD	% Purity (*w*/*w*)
Oleuropein	0.602	0.010	0.01	60.8 ± 0.27
α-Tomatine	0.953	0.052	5.42	94.2 ± 0.49

## Data Availability

Not applicable.

## Supplementary Materials

The following supporting information can be downloaded at: https://www.mdpi.com/article/10.3390/ijms23137424/s1.
