# Supplementary material for: Bioactive Compounds and Their Impact on Protein Modification in Human Cells
Source: Int J Mol Sci. 2022 Jul 4;23(13):7424. doi: 10.3390/ijms23137424 (PMC9266987; doi:10.3390/ijms23137424)
Supplement: Supplementary file 1 [file ijms-23-07424-s001.zip › ijms-1756369-supplementary.pdf]

Supplementary data S1

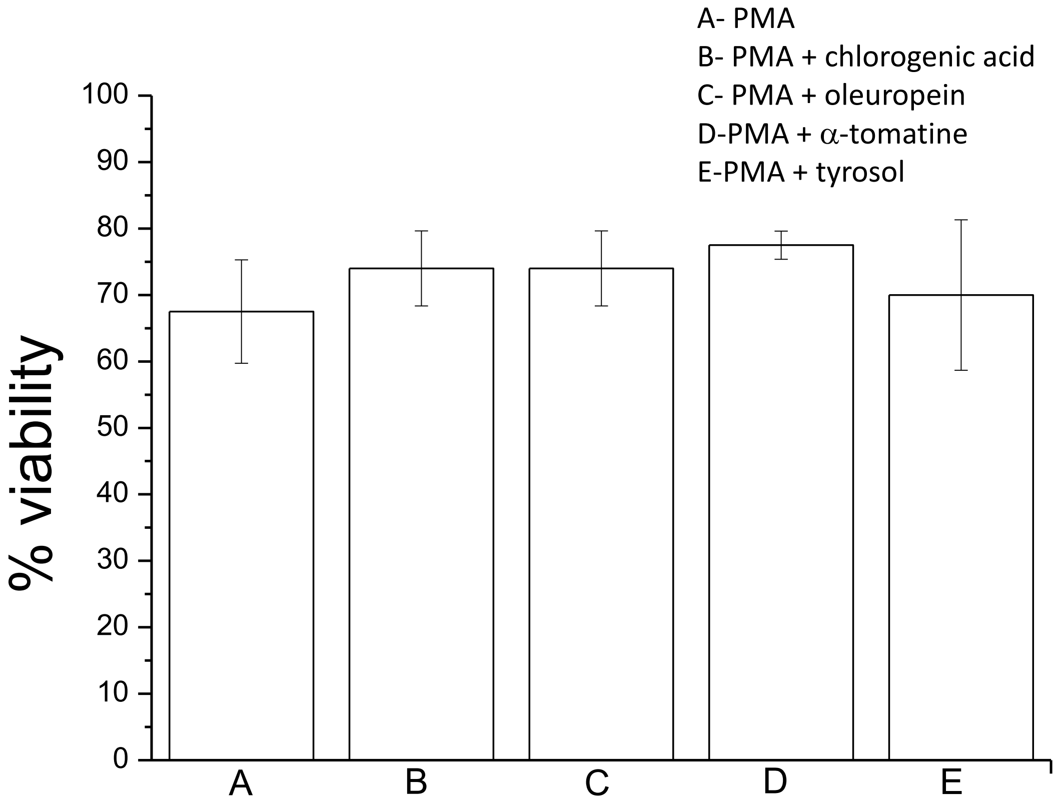

Supplementary data S1: Effect of PMA (250 nM) (A) and in presence of chlorogenic acid (B), oleuropein (C), α-tomatine (D) and tyrosol (E) on viability of U937 cells. The viability was measured in 72h differentiated U-937 celsl with last 24 h of incubation with bioactive compounds. Trypan blue (0.1%) was mixed in the ratio 1:1. The ratio of alive:dead cell was measured using a counting slide (dual chamber for cell counter, Bio-Rad) on an automated cell counter and %viability is plotted.

Supplementary data S2

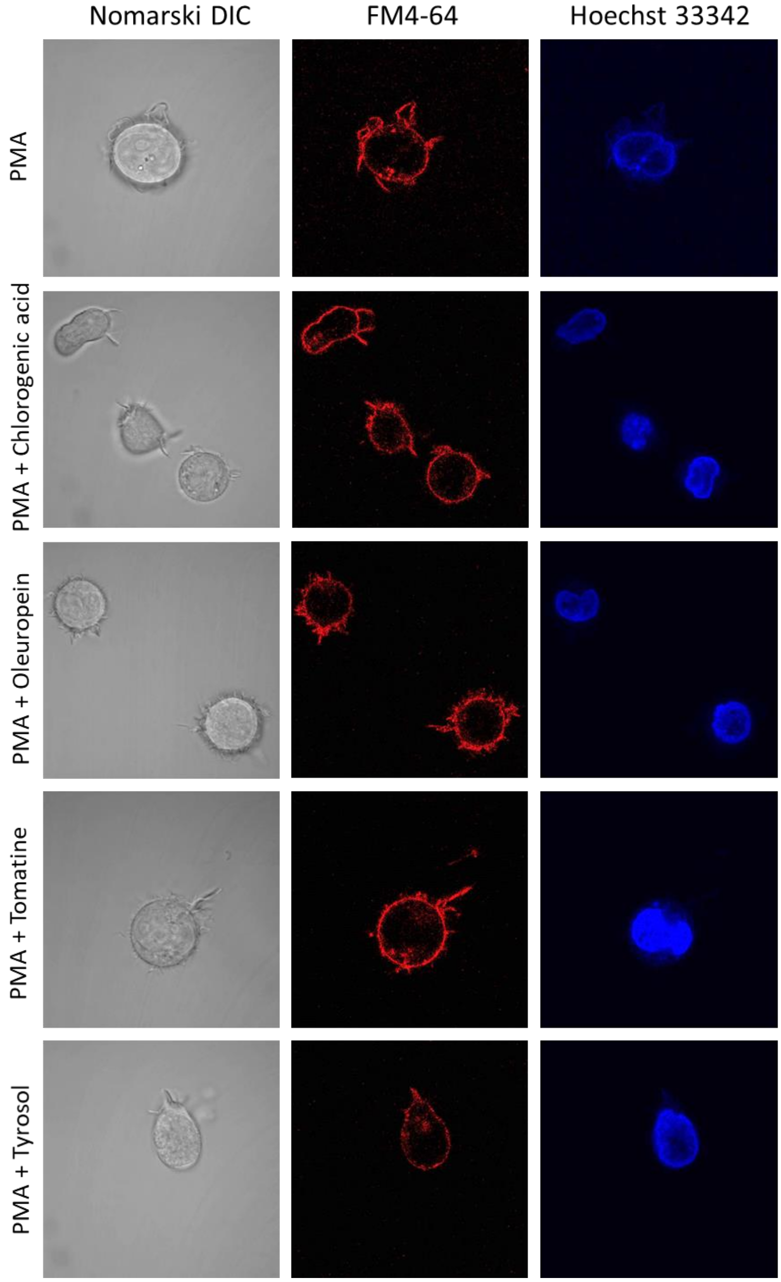

Supplementary data S2: Double staining with Hoechst 33342 and FM4-64 in 48 h differentiated U937 cells and in the presence of either of the bioactive compounds (chlorogenic acid, oleuropein, α-tomatine and tyrosol, 10μM). The staining was done for 5 min and images were taken in different channels (from left to right are Nomarski DIC, FM4-64 and Hoechst 33342).
